# Differential Expression of Genes Related to Sexual Determination Can Modify the Reproductive Cycle of *Astyanax scabripinnis* (Characiformes: Characidae) in B Chromosome Carrier Individuals

**DOI:** 10.3390/genes10110909

**Published:** 2019-11-08

**Authors:** Jonathan Pena Castro, Ricardo Shohei Hattori, Túlio Teruo Yoshinaga, Duílio Mazzoni Zerbinato de Andrade Silva, Francisco J. Ruiz-Ruano, Fausto Foresti, Mateus Henrique Santos, Mara Cristina de Almeida, Orlando Moreira-Filho, Roberto Ferreira Artoni

**Affiliations:** 1 Departamento de Genética e Evolução, Programa de Pós-Graduação em Biologia Evolutiva e Genética Molecular, Universidade Federal de São Carlos, Rodovia Washington Luis, Km 235, Monjolinho, São Carlos, SP 13565-905, Brazil; omfilho@ufscar.br (O.M.-F.); rfartoni@gmail.com (R.F.A.); 2 Estação Experimental de Salmonicultura de Campos do Jordão, UPD-CJ (APTA/SAA), Campos do Jordão, São Paulo, SP 12460-000, Brazil; hattori.fish@gmail.com; 3 Faculdade de Medicina Veterinária e Zootecnia da Universidade de São Paulo, Departamento de Cirurgia, Universidade de São Paulo, Butantã, Rua Professor Orlando Marque Paiva, São Paulo, SP 05508-270, Brazil; teruo92@gmail.com; 4 Departamento de Morfologia, Instituto de Biociências, Universidade Estadual Paulista, Distrito de Rubião Junior, s/n, Botucatu, SP 18618-970, Brazil; duiliozerbinato@gmail.com (D.M.Z.d.A.S.); fforesti@ibb.unesp.br (F.F.); 5 Department of Ecology and Genetics, Evolutionary Biology Centre, Uppsala University, SE-752 36 Uppsala, Sweden; francisco.ruiz-ruano@ebc.uu.se; 6 Departamento de Biologia Estrutural, Molecular e Genética, Programa de Pós-Graduação em Biologia Evolutiva, Universidade Estadual de Ponta Grossa, Avenida Carlos Cavalcanti, 4748, Ponta Grossa, PR 84030-900, Brazil; mateushenrique@yahoo.com.br (M.H.S.); almeidamara@uol.com.br (M.C.d.A.)

**Keywords:** fishes, supernumerary chromosomes, sexual differentiation, *foxl2a*, *amh*

## Abstract

The species complex *Astyanax scabripinnis* is one of the most studied with respect to origin, distribution, and frequency of B chromosomes, and is considered a model organism for evolutionary studies. Research using population inferences about the occurrence and frequency of the B chromosome shows seasonal variation between sexes, which is associated with the presence of this supernumerary element. We hypothesized that the B chromosome could influence the sex ratio of these animals. Based on this assumption, the present work aimed to investigate if differences exist among levels of gene expression with qRT-PCR of the *amh* (associated with testicular differentiation) and *foxl2a* (associated with ovarian differentiation) genes between B-carrier and non-B-carrier individuals. The results showed that for the *amh* gene, the difference in expression between animals with B chromosomes was not accentuated compared to that in animals without this chromosome. Expression of *foxl2a* in B-carrier females, however, was reduced by 73.56% compared to females that lacked the B chromosome. Males had no difference in expression of the *amh* and *foxl2a* genes between carriers and non-carriers of the B chromosome. Results indicate that the presence of B chromosomes is correlated with the differential expression of sex-associated genes. An analysis of these results integrated with data from other studies on the reproductive cycle in the same species reveals that this difference in expression may be expanding the reproductive cycle of the species.

## 1. Introduction

The process that shapes sexual evolution and sex determination is one of the most interesting and fundamental aspects of the history of life. The sex development of several vertebrate groups has been described, which allows for the establishment of two mechanisms of sex determination: genetic (GSD) and environmental sex determination (ESD) [1]. In fish, genetic sex determination can be triggered primarily through the action of a master gene or various loci associated with sex, while environmental sex determination is regulated by external factors (e.g., water temperature), and a mixture of both mechanisms may act in sexually undifferentiated gonads [2]. However, in subsequent events, a shift can occur during sexual differentiation, such as sex inversion, where genotypic females and males develop as phenotypic males and females, respectively [3].

For a long time, the knowledge of sex determination in fish was associated with homologous genes related to sex development usually found in other species or more distant groups. Currently, some master genes of sex determination have been described related to male development, such as *dmy* (Y-specific DM-domain) in *Oryzias latipes*, *gsdfy* (gonadal soma-derived growth factor on the Y chromosome) in *Oryzias luzonensis*, and *sdy* (sexually dimorphic on the Y chromosome) in salmonids [4,5,6]. In addition, a second copy of the anti-Müllerian hormone gene, *amhy* (Y-linked anti-Müllerian hormone) has been described as a sex determinant gene in *Odontesthes hatcheri*, *Odontesthes bonariensis*, and *Hypoatherina tsurugae* [7,8,9]. Although fish do not have Müllerian ducts, elevated levels of *amh* expression were detected in the early stages of testis development in several teleosts [10,11,12]. Furthermore, in *Oreochromis niloticus*, *amh* was expressed at low levels in gonads before sexual differentiation in both XX and XY animals, and also presented dimorphic expression in males during germinative lineage differentiation [13], and a recent study reported a second copy of *amh* linked with the Y chromosome that might be a candidate as a sex determinant gene in Nile tilapia [14]. This data suggests that *amh* genes are strongly related to male gonad development in several fish species.

On the other hand, the transcription factor *forkhead box like-2 (foxl2a)* is associated with ovarian differentiation and the development of conserved features among vertebrates [13]. *The foxl2* gene is the earliest detected gene expressed during ovarian development in mammals and other vertebrates. In mammals, *foxl2* promotes ovarian development by upregulating aromatase expression, hence inducing estrogen production [15]. This mechanism has also been described in many teleosts. In addition, at least two isoforms have been described in several fish species (*foxl2a* and *foxl2b*) [16], such as Nile tilapia [17], zebrafish [18], and rainbow trout, supporting the conserved role of *foxl2* during ovary development from fish to mammals.

Although functions related to *amh* and *foxl2* have been described in many fish species, their role in sex development and sex determination in neotropical fish models remains unknown. Neotropical fish are a special group with more than 5000 known species [19]. Among them, the *Astyanax* genus (Baird and Girard, 1854) comprises a dominant group in South America, including at least 250 known species [20,21], *Astyanax scabripinnis* being one of the most studied [22]. Furthermore, a remarkable cytogenetic feature is the presence of B chromosomes equivalent in size to the first pair of karyotype complement in different allopatric populations [23]. In most populations, only one B chromosome per metaphase is consistently found in individuals, and all individuals carry exactly the same B chromosome [24]. Individuals carrying two B chromosomes are extremely rare [23].

The B chromosome comprises an additional genetic element found in all eukaryote groups, which do not pair with chromosomes of standard complement (chromosome A) during meiosis, exhibiting an irregular segregation that does not obey the Mendelian principles underlying the mechanisms of population accumulation [25]. Until recently, it was believed that B chromosomes did not carry functional genes and were not able to change the phenotype of the carrier organisms [25,26,27]. However, genes related to cellular division, the mitotic cycle, cellular metabolism, and nucleotide transcription, in which B chromosome transcripts were correlated in mammals and invertebrates, were recently reported [28,29,30]. In fish, B chromosome carrier males of *A. scabripinnis* presented higher levels of *dmrt1* expression during the maturation stage [31]. In addition, a correlation of the sex ratio with B chromosome carrier individuals of *A. scabripinnis* was made, where seasonal distortions increased the sex ratio in favor of females [32,33,34,35]. Moreover, B-carrying females demonstrated a delay in the reproductive peak, suggesting a possible adaptive role of the B chromosome in the reproductive cycle [35]. 

Thus, based on previous studies, we hypothesized that B chromosome occurrence could influence the reproductive cycle of *A. scabripinnis* populations through the regulation of sex-development-related genes such as *amh* and *foxl2a*. Thus, our aim was to characterize the expression of *amh* and *foxl2a* genes between males and females and their relation to the presence of the B chromosome in *A. scabripinnis* individuals.

## 2. Materials and Methods

### 2.1. Characterization of the Study Object

We sampled 24 adult specimens (12 females, 12 males) of *A. scabripinnis* from Guaratinguetá (Fazenda Lavrinha), Estado de São Paulo, Brasil (22°40′49.5″ S, 45°23′31.9″ W). They were captured actively with 0.5 mm mesh sieves and passively with covo-type traps during their reproductive cycle (November to January). Captured fish were conditioned in plastic bags with water and oxygen under pressure, then transported to the Estação Experimental de Salmonicultura (APTA/UDC—Campos do Jordão, São Paulo) for processing. All procedures were conducted in accordance with the guidelines of animal experimentation established by the Comissão de Ética em Pesquisa Animal da Universidade Estadual de Ponta Grossa (COEP-UEPG) with legal authorization provided by the Instituto Chico Mendes de Conservação da Biodiversidade (ICMBio—# 15115-1).

### 2.2. Cytogenetics 

Identification of animals with the B chromosome was achieved using the techniques described by Bertollo et al. [36]. Briefly, a microdissected B chromosome probe was amplified through a degenerate oligonucleotide primer. PCRs were performed with 1× Taq DNA Polymerase Buffer (Invitrogen), 2 mM of MgCl_2_, 40 µM of dTTP, dGTP, and dCTP, 20 µM of dATP, 20 µM of digoxigenin-16-dUTP, 2 µM of DOP primer, and 2U of Taq DNA polymerase. Amplification was performed under the following conditions: 3 min at 94 °C, 35 cycles of 90 s at 90 °C, 90 s at 52 °C, and 90 s at 72 °C; followed by a post-cycling extension for 5 min at 72 °C. The obtaining of mitotic chromosomes and fluorescent in situ hybridization (FISH) with a whole chromosome painting B probe was carried out according to Vicari et al. [37] and Cornelio et al. [35]. Hybridization occurred under high-stringency conditions (2.5 ng/µL probe, 50% formamide, 2×·SSC, 10% dextran sulfate). Signals were detected with the Anti-Digoxigenin-Rhodamine antibody (Roche, Mannheim, Germany). Materials were counterstained with ProLong Gold Antifade Mountant with DAPI (Thermo Fisher, Burlington, ON, Canada). Preparations were analyzed using an epifluorescence microscope (Olympus BX41, New York, NY, USA) coupled to an image capture system (CCD Olympus DP 71 and DP Controller v. 3.2.1.276).

### 2.3. Histological Analysis

Histological procedures were performed to confirm the individual sex and classification of the maturation stage of gonadal development. Gonad samples were dissected and fixed in Bouin solution for 24 h at room temperature. Samples were subsequently dehydrated, embedded in paraffin, sliced into sections of 3–5 µm thickness, and stained with hematoxylin and eosin. The gonads were classified in the mature state, based on the development stage, according to Vazzoler [38].

### 2.4. RNA Extraction and cDNA Synthesis 

All the animals were kept in the same acclimation conditions in the laboratory (250 L tanks with constant aeration, controlled temperature at 25 °C, 12 h light, and fed once a day for 48 h) until tissue sample collection. After dissection, gonads were immediately fixed in RNAlater Solution (Ambion, Vilnius, Lithuania). Total RNA was extracted through homogenization in TRIzol (Invitrogen, Carlsbad, CA, USA) according to the manufacturer’s instructions and purified with DNase I, Amplification Grade (Invitrogen). Total RNA concentration was measured by spectrophotometry and the integrity was verified by electrophoresis of 28S and 18S bands in 1% agarose gel. The cDNA was synthesized with 1 µg of total RNA using Oligo(dT)_12–18_ Primer (Invitrogen) and reverse transcriptase was performed using SuperScript III (Invitrogen).

### 2.5. Quantitative PCR in Real-Time (qRT-PCR)

We designed specific primers of the *amh* and *foxl2* genes for qRT-PCR analysis in *A. scabripinnis*. First we got the sequences of *amh* (ENSAMXG00000032908) and *foxl2a* (ENSAMXG00000026282), amplifying by conventional PCR with primers designed from conserved regions (determined by comparison with the sequences of other fish species, i.e., *Pygocentrus nattereri*, *Danio rerio*, *Ictalurus punctatus*, *Scleropages formosus*, *Cyprinus carpio*) of the *Astyanax mexicanus* genome assembly (Accession GCA_000372685.1) by searching in the Ensembl database (http://www.ensembl.org/index.html). These sequences were the exon–exon junctions from the CDS (coding sequence) regions (Table 1). PCR reactions contained 1× PCR buffer, 1.5 mM MgCl_2_, 200 µM dNTPs, 0.1 µM of each primer (10 pmol), 0.5 U Taq DNA Polymerase (Invitrogen), and 1 µL (100 ng) of *A. scabripinnis* genomic DNA (obtained by saline extraction method, according to the protocol of Bruford [39]). We performed PCR amplification starting with 95 °C for 5 min, followed by 35 cycles at 94 °C for 1 min, 51 °C for 45 s, and 72 °C for 1 min and 20 s, and a final extension of 72 °C for 5 min. Products were purified using the High Pure PCR Cleanup Micro Kit (GE Healthcare Amersham Biosciences, Buckinghamshire, UK) and sequenced using an ABI PRISM^™^ 377 DNA Sequencer (Perking-Elmer, Foster City, USA) with the DYEnamic^™^ ET Terminator Cycle Sequencing Kit (GE Healthcare Amersham Biosciences, Buckinghamshire, UK). We followed the manufacturer’s instructions for both steps. After obtaining the sequences, *A. scabripinnis* specific primers were designed for qRT-PCR (Table 1). The entire procedure of alignment, primer construction, and BLASTn search was performed in Geneious R11’s suite of molecular biology and NGS analysis tools.

The number of individuals utilized in each sample group is summarized in Table 2. The qRT-PCR was performed in the thermocycler Stratagene Mx3005P (Agilent Technologies, Waldbronn, Germany) using SYBR Select Master Mix (Life, Vilnius, Lithuania) in a final volume of 12.7 µL, with 2 µL of cDNA in each reaction and 0.4 µL of each primer (10 mM). The thermal profile was 95 °C for 10 min, followed by 40 cycles at 95 °C for 40 s, and 60 °C for 1 min for both genes. After amplification, the dissociation step was realized by increasing the temperature from 60 °C to 95 °C in order to create a melting curve and ensure the presence of only the amplification product. Samples without Ct values or with inconsistent Ct values between replicas (difference Ct > 2 cycles) were excluded from the analysis.

The qPCR data were obtained by MxPro software (Agilent Technologies) and normalized against values of β-actin and the quantification obtained by a 2^−ΔΔCT^ method [40]. The values were submitted to the normality test (Shapiro–Wilk normality test) and homoscedastic variance analysis (Breusch–Pagan test). The qPCR values were presented as the mean ± standard error of the mean (SEM) and analyzed by ANOVA one way, the Student’s *t*-test, and Tukey’s test with significant values at *p* < 0.05 (Supplementary Table S1), in GraphPad Prism 7 software. 

### 2.6. Illumina Sequencing and Coverage Analysis of Sex-Associated Genes

The presence of *amh* and *foxl2a* genes in the *A. scabripinnis* B chromosome was determined by comparing the coverage patterns between three individuals without B and another three with B in gDNA Illumina HiSeq X Ten libraries, paired-end reads of 2× 150 nt. The genome coverage was calculated assuming that the genome size of *A. scabripinnis* is 1.87 Gb (Table 3) [41]. As reference for the *amh* and *foxl2a* genes, we used the sequences in *A. mexicanus* with Genbank accession numbers XM_022669774 and XM_007232295, respectively. First, we extracted similar reads from the CDSs and reconstructed their sequences specifically to *A. scabripinnis* using customized scripts (github.com/fjruizruano/whatGene/blob/master/scripts/mapping_blat_gs.py, option nomap) with the option of considering mapping of at least 40 nt with a minimum identity of 80%. Then, we mapped the reads of each library on the CDSs through the SSAHA2 software [42] with a minimum alignment score of 40 and 80% minimum identity and obtained the abundance estimate in each library by counting the reads mapped by a custom script (github.com/fjruizruano/whatGene/blob/master/scripts/mapping_blat_gs.py, option ssaha2). Finally, we estimated the abundance per nucleotide position for each CDS in the B− and B+ genomes using a custom script (github.com/fjruizruano/ngs-protocols/blob/master/bam_coverage_join.py) and graphics were made with a custom script (github.com/fjruizruano/whatGene/blob/master/scripts/coverage_graphics.py).

## 3. Results 

### 3.1. Histological Analysis and Cytogenetics

**Figure 1:**
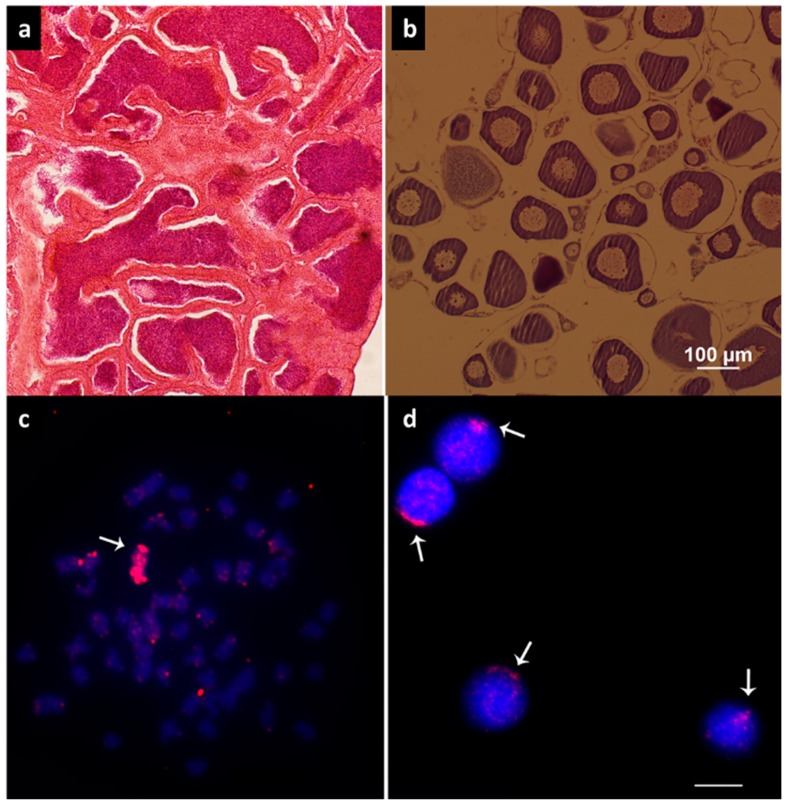
Histological sections of *A. scabripinnis* gonads. (**a**) Male. (**b**) Female. (**c**) Fluorescent in situ hybridization (FISH) with B chromosome probe in metaphasis and (**d**) nuclei, respectively. Arrows indicate the B chromosome probe signal. Bar: 10 µm.

Histological analysis revealed the phenotypic sex and gonad maturation stages of the analyzed individuals (Figure 1a,b). In situ hybridization revealed the presence of the B chromosome in three females of *A. scabripinnis*, while seven males were observed with an occurrence of the B chromosome (Figure 1c,d).

### 3.2. Analysis of Gene Expression of Amh and Foxl2a

**Figure 2:**
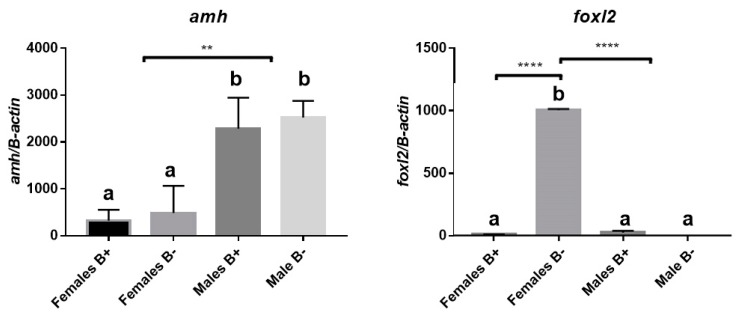
Relative expression levels of *amh* and *foxl2a* of adult *A. scabripinnis*. Groups that are significantly different from each other are represented by different letters (a and b). (B+) individuals with and (B−) without the B chromosome, respectively. **** *p* < 0.0001, extremely significant; ** *p* 0.001 to 0.01, very significant.

Gonads of analyzed individuals showed significant bimodal expression (*p*-value < 0.05) for the *amh* gene between males and females, independent of the presence of the B chromosome (Figure 2, Supplementary Table S1). Samples of gonadal tissue were analyzed for *foxl2a* gene expression. This analysis revealed significant differences of bimodal expression between females with B chromosomes and other groups. The number of females with B chromosomes was reduced by 73.56% compared to females that lacked the B chromosome (Figure 2, Supplementary Table S1).

### 3.3. Number of Gene Copies between Individuals with and without B Chromosomes

**Figure 3:**
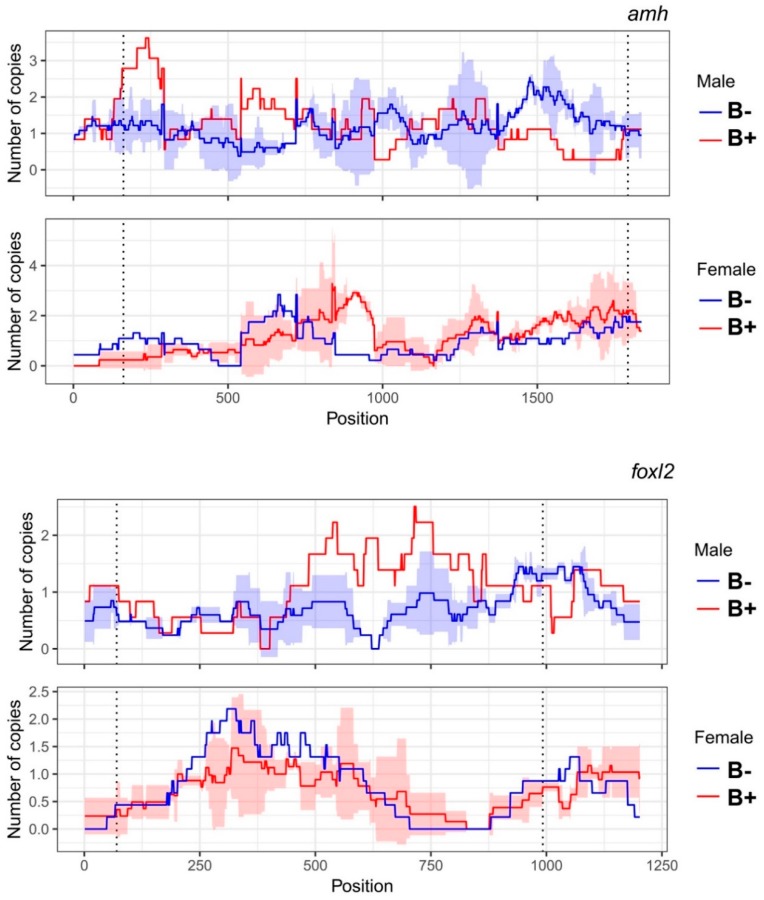
Comparison of the number of copies of the *amh* and *foxl2a* genes in gDNA of individuals with (B+) and without the B chromosome (B−). The dotted line is the limit of the coding sequence (CDS). The *p*-value (*p* < 0.05) between copy number was not significantly different (*p* = 0.8509 and 0.7333 for the *t*-test of male and female *amh*, respectively, and *p* = 0.2979 and 0.7752 for the *t*-test of male and female *foxl2a,* respectively).

The coverage analysis of libraries B− and B+ along with CDSs of the *amh* and *foxl2a* genes revealed no differences between groups in any of the genes analyzed (Figure 3). Although the graph shows some regions with differences in coverage between groups, there is clearly no uniformity. Thus, we infer that these genes are not present in the B chromosome of this population. 

## 4. Discussion

Neotropical fish species comprise an interesting group with a considerable variety of physiological and reproductive mechanisms. In addition, some species such as *A. scabripinnis* have the B chromosome, a remarkable cytogenetic characteristic that recently demonstrated roles in diverse cellular functions. This study is a pioneer in analyzing the expression of *amh* and *foxl2a* genes in male and female gonads of fish and the influence of the B chromosome’s presence. Here we demonstrated that the *amh* gene presented a dimorphic expression between females and males of *A. scabripinnis* independent of the presence of the B chromosome. Furthermore, *foxl2a* also presented a dimorphic expression between females and males, but B+ females presented a lower expression of *foxl2a* compared to a B− female, suggesting a regulatory effect of the B chromosome in *foxl2a* expression during the reproductive period in B+ females.

The higher expression of *amh* in males compared to females is consistent with other reported studies. *Amh* plays important roles in sex determination, testis development during embryonic stages, and spermatogenesis in many teleost fishes [43]. *Amh* function in fish is supposed to act as an aromatase inhibitor like it does in mammals, hence inhibiting estrogen production during testis development and spermatogenesis [7]. However, this relation is not well established since other studies have demonstrated independent expression of aromatase and *amh* in other species [11,44,45]. Nevertheless, second copies of the *amh* gene were not detected in the B chromosome of *A. scabripinnis* and their expression did not differ between B+ or B− males.

Although the B chromosome apparently did not affect *amh* expression, this was not the case with *foxl2a*. B+ females presented an atypical expression of *foxl2a* compared to a B− female. *Foxl2a* has been associated with ovarian development in all studied species (revisited in Bertho et al. [16]), demonstrating clearly high expression in females compared to males, which is conserved condition for this gene among teleost fishes [13]. Nevertheless, although other copies of *foxl2a* were not found in B+ sequenced females, other factors might regulate gene expression. Some studies suggest transcription regulation of A chromosome genes by non-coding mRNA in the B chromosome in cichlid fish and insects [46,47]. In addition, previous studies reported over-expression of the *dmrt1* gene expression in B+ male during the maturation stage [31]. Thus, the B chromosome may have some regulatory role on *foxl2a* expression in *A. scabripinnis* females, and such regulatory effect of the B chromosome on sex development genes could be responsible for the uncharacteristic reproductive period of B+ individuals, and also the disproportional sex ratio reported [32,34,48,49].

**Figure 4:**
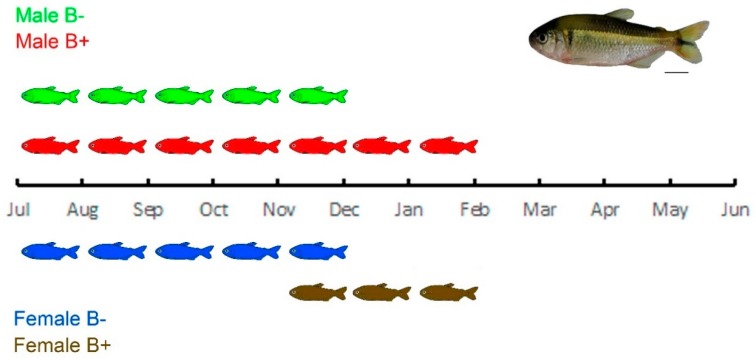
Representation and data of the reproductive period of *A. scabripinnis,* according to [31,35]. B−: without B chromosome, B+: with B chromosome. It is important to highlight the late reproductive activity of females with B chromosomes and the extension of males carrying the additional chromosome, which allows them to reproduce with females with or without B chromosomes throughout the period, ensuring the maintenance of this chromosome in the population. Fish bar: 1 cm.

Studies on the reproductive cycle in this species demonstrated that B− females have reproductive activity from July to December, while B+ females present a reproductive peak shorter and later, from December to February [35]. This regulatory effect could cause, in the case of females, later ovary development, since B+ females presented lower expression of *foxl2a* compared to a B− female, and extending male spermatogenesis due to the upregulatory effect on *dmrt1* expression reported for B+ males of *A. scabripinnis* [31] (Figure 4). Such an extended reproductive period may contribute to the maintenance of this chromosome and consist of an important adaptive advantage that maintains populations in small high-altitude streams, which are constantly susceptible to intense environmental variations and predation [35,50,51]. Regarding a disproportional sex ratio, our sampling did not demonstate such a difference, mainly because we chose not to do that. Nevertheless, the B chromosome could also regulate sex development genes during embryonic stages, increasing the number of females. However, the sex ratio can be altered by environmental factors or endocrine disruptors, as demonstrated in several fish species, and the B chromosome is likely not involved [52,53].

In summary, these results highlight that the presence of the B chromosome significantly alters the expression profile of major sex-linked genes, indicating the adaptive relevance of this chromosome, directly influencing the reproductive cycle dynamic of these animals and expanding the reproduction period, enabling the generation of a greater number of offspring. Although we did not detect second copies of analyzed genes in the B chromosome, regulatory effects could occur in B-carrier animals through non-coding mRNA, transposons, or even microRNAs. However, how the B chromosome regulates these genes in *A. scabripinnis* requires further investigation, and novel techniques with B-omics will help us understand the genetic, transcriptional, epigenetic, and protein functions involved in cellular biology and their relation to sex development [28].

## Figures and Tables

**Table 1:** Designed primers used for conventional PCR and qRT-PCR.

Gene	Primer Sequence (5′–3′)	Use
*ame_amh_Fw1*	CTGGGATGTTGAAGACGA	Conventional PCR
*ame_amh_Rv1*	GAGGAATTAATCAGCTCCAGAA	Conventional PCR
*ame_fox2_Fw1*	ACGTTCTTGGGCTCAGAGGA	Conventional PCR
*ame_foxl2a_Rv1*	AGACTTGCCGGGTTGGAAGTG	Conventional PCR
*amh_rtf1*	CCTCACTGCTCTTCCTGACG	qRT-PCR
*amh_rtR1*	AAACACCCAACACAGCTTGC	qRT-PCR
*foxl2a_rtF1*	ACCTGAGCCTTAACGAGTGC	qRT-PCR
*foxl2a_rtR1*	ATGTCTTCACACGTCGGGTC	qRT-PCR
*β-actin F_RT*	ATCATGAAGTGCGACGTGGA	qRT-PCR
*β-actin R_RT*	TATTTACGCTCAGGTGGGGC	qRT-PCR

**Table 2:** Individual samples used in gene expression analysis.

Gene	♀ (B+)	♀ (B−)	♂ (B+)	♂ (B−)	Total
*amh*	3	3	3	3	12
*foxl2a*	3	3	3	3	12
**Total**					24

**Table 3:** Genome coverage and samples of Illumina sequencing.

Sex	Samples	Number of B	Number of Reads (mi)	Coverage
**Male**	2	0	93.7	7.5
	1	1	52.2	4.2
**Female**	1	0	43.7	3.5
	2	1	96	7.7

## Supplementary Materials

The following are available online at https://www.mdpi.com/2073-4425/10/11/909/s1: Table S1: Statistical analysis (*p <* 0.05) for *amh* and *foxl2a* genes.
