# Supplementary material for: Differential Expression of Genes Related to Sexual Determination Can Modify the Reproductive Cycle of Astyanax scabripinnis (Characiformes: Characidae) in B Chromosome Carrier Individuals
Source: Genes (Basel). 2019 Nov 8;10(11):909. doi: 10.3390/genes10110909 (PMC6896079; doi:10.3390/genes10110909)
Supplement: Supplementary file 1 [file genes-10-00909-s001.pdf]

Supplementary Table S1. Statistical analysis (*p < 0.05*) for *amh* and *foxl2a* genes.

amh

Shapiro–Wilk normality test	Females B+	Females B-	Males B+	Males B-
W	0.999	0.8987	1	1
p-Value	0.9402	0.3813	>0.9999	>0.9999
Passed normality test (alpha = 0.05)?	Yes	Yes	Yes	Yes
p-Value summary	ns	ns	ns	ns
**Test for homoscedasticity**				
Rs of Predicted Y vs. abs(residual)	−0,5	0,5	−0,5	0
p-Value (one tailed)	0.5000	0.5000	0.5000	0.6667
Passed (*p* > 0.05)?	Yes	Yes	Yes	Yes

Tukey's multiple comparisons test	Mean Diff,	95.00% CI of diff,	Summary	Adjusted *p*-Value	Significant?
Females B+ vs. Females B-	–159.3	–1436 to 1117	ns	0.9770	No
Females B+ vs. Males B+	–1961	–3237 to –683.9	**	0.0051	No
Females B+ vs. Male B-	–2197	–3473 to –920.1	**	0.0025	Yes
Females B- vs. Males B+	–1801	–3078 to –524.7	**	0.0084	Yes
Females B- vs. Male B-	–2037	–3314 to –760.9	**	0.0040	Yes
Males B+ vs. Male B-	–236.2	–1513 to 1040	ns	0.9316	No

B+, individual with B chromosome; B-, individual without B chromosome; ns, not significant; **** $p < 0.0001$, extremely significant; *** $p = 0.0001$ to 0.001, extremely significant; ** $p = 0.001$ to 0.01, very significant.

foxl2a

Shapiro–Wilk normality test	Females B+	Females B-	Males B+	Males B-
W	0.992	1	1	0.9193
p-Value	0.8289	>0.9999	>0.9999	0.4500
Passed normality test (alpha = 0.05)?	Yes	Yes	Yes	Yes
p-Value summary	ns	ns	ns	Ns
**Test for homoscedasticity**				
Rs of Predicted Y vs. abs(residual)	−0.5	0.5	-0.5	0.5
p-value (one tailed)	0.5000	0.5000	0.5000	0.5000
Passed (*p* > 0.05)?	Yes	Yes	Yes	Yes

Tukey's multiple comparisons test	Mean Diff	95.00% CI of diff	Summary	Adjusted *p*-Value	Significant?
Females B+ vs. Females B-	–999.6	–1041 to –958.4	****	<0.0001	Yes
Females B+ vs. Males B+	–15.04	–56.28 to 26.2	ns	0.6618	No
Females B+ vs. Male B-	10.09	–31.15 to 51.33	ns	0.8599	No
Females B- vs. Males B+	984.6	943.3 to 1026	****	<0.0001	Yes
Females B- vs. Male B-	1010	968.5 to 1051	****	<0.0001	Yes
Males B+ vs. Male B-	25.13	–16.11 to 66.37	ns	0.2811	No

B+, individual with B chromosome; B-, individual without B chromosome; ns, not significant; **** $p < 0.0001$, extremely significant; *** $p = 0.0001$ to 0.001, extremely significant; ** $p = 0.001$ to 0.01, very significant.
